# *Micromeria barbata* for targeting MRSA virulence: *In silico* and *in vitro* studies

**DOI:** 10.1016/j.heliyon.2024.e41536

**Published:** 2024-12-29

**Authors:** Shiraz Rawas, Nawal Al Hakawati, Amale Mcheik, Dalia El S El Badan

**Affiliations:** a Department of Biological Sciences, Faculty of Science, Beirut Arab University, Tripoli, 1300, Lebanon; b Department of Natural Sciences, School of Arts and Sciences, Lebanese American University, P.O.Box 13–5053, Chouran Beirut, 1102 2801, Beirut, Lebanon; c Botany and Microbiology Department, Faculty of Science, Alexandria University, Alexandria, Egypt

**Keywords:** *Micromeria barbata*, Ethanolic crude extract, Crude characterization, Antimicrobial activity

## Abstract

The present study reports the characterization of the phytochemical content and the antibacterial activity of ethanolic extracts from the leaves (LE) and stems (SE) of *Micromeria barbata (M.barbata)* against Methicillin resistant *Staphylococcus aureus* (MRSA*)*. Important functional groups were determined by analyzing the FTIR spectra of LE and SE. The phytochemical profiles were analyzed by GC-MS, and these characterized the chemicals according to retention periods and peak regions. The binding affinities of discovered compounds with the MRSA SarA and CrtM proteins were evaluated using molecular docking approach. Assays for biofilm formation, MIC, MBC, and agar well diffusion were used to assess the antimicrobial activity. From the FTIR spectra, hydroxyl group alkanes, amides, and aromatic constituents were identified. Nine distinct substances were obtained from GC-MS analysis;, including piperitenone cyclohexene, palmitic acid, safranal, and oleic acid in SE, and iso-menthone, oleic acid, 1-pentanol, kumarone, and benzene in LE. Stronger binding affinities between LE compounds and the SarA protein were obtained from molecular docking with ΔG values ranged from −10.9 to −11.6 were higher compared to SE compounds. Antimicrobial findings showed that SE and LE exhibited an antimicrobial effect against *S. aureus* with 15 and 20 mm respectively as inhibition zones. MIC values for LE and SE were 6.25 mg/ml and 12.5 mg/ml, respectively. With LE showing greater effectiveness, both extracts exhibited concentration-dependent reduction of biofilm formation and bactericidal activity. Extracts of *M. barbata*, notably the leaves, have significant antibacterial activity against *S. aureus*, suggesting potential applications in the developing of novel antibacterial treatments.

## Abbreviation

SE: Ethanolic Stem crude
LE: Leaves Ethanolic crude
MHB: Muller Hinton Broth

## 1 Introduction

Methicillin-resistant *Staphylococcus aureus* (MRSA) stands as well -known pathogen, posing significant clinical challenges due to its resistance to methicillin and other β-lactams, along with its ability to resist multiple antimicrobial agents. Its impact is particularly shown in hospital settings, where it infects vulnerable patients with predisposing conditions such as surgical wounds, indwelling catheters, immunosuppression, and severe underlying diseases [4]. The burden of MRSA infections is amplified by increased costs stemming from the necessity for more expensive treatments, prolonged hospital stays, and heightened rates of treatment failure compared to infections caused by methicillin-sensitive *S. aureus* (MSSA).

Despite its historical association with healthcare facilities, MRSA has increasingly encroached upon the community setting, where it afflicts otherwise healthy individuals, causing a spectrum of infections ranging from skin and soft tissue infections (SSTIs) to life-threatening invasive diseases like necrotizing pneumonia and osteomyelitis. Compounding the challenge, both hospital-acquired and community-acquired MRSA infections exhibit a problematic high rate of recurrence, particularly evident in SSTIs [17].

Understanding the determinants of MRSA-specific diseases and the intricate host-pathogen interactions during MRSA infection is paramount for developing effective prevention and treatment strategies [5]. One significant hurdle in managing MRSA infections is the development of biofilms, which substantially heightens the risk of morbidity and mortality in clinical settings. Biofilm formation is tightly regulated by various mechanisms, including two-component systems (TCS) such as agr, nucleic acid-binding proteins like SarA, and the production of virulence factors like staphyloxanthin [10,19,29].

Central to MRSA virulence is diapophytoene synthase, also known as CrtM, which is regulated by SarA, a transcription factor pivotal in modulating the expression of numerous virulence genes. Disruption of SarA function can impede the transcription of critical messenger RNAs, impacting the pathogen's virulence [14,21]. Additionally, CrtM contributes to pathogenesis by facilitating the synthesis of staphyloxanthin, which provides protection against oxidative stress [28].

Given the close connection between virulence factors and biofilm formation, targeted strategies to hinder MRSA biofilm development have emerged as promising avenues for infection control [26]. Recent research underscores the potential of natural antibiofilm agents in thwarting biofilm formation and disrupting existing ones by suppressing virulence factor expression in bacteria [22]. Notably, these agents exhibit a unique characteristic of not impeding bacterial metabolic growth, reducing the likelihood of resistance development [27].

In this context, bioactive compounds derived from natural sources, including plants and animals, have garnered attention as crucial contributors to drug development efforts. Micromeria, a genus within the Lamiaceae family, has emerged as a particularly promising source of such compounds. With over 78 acknowledged species, Micromeria, notably *Micromeria barbata*, has been recognized for its diverse chemical profiles, particularly rich in essential oils with pharmacological properties such as antimicrobial, antioxidant, anticancer, and antidepressant effects [15].

The present study focuses on the identification of phytochemical compounds of the ethanolic crude extract of *M. barbata* leaves and stem using GC-MS and FTIR analyses. Antimicrobial activity of the extracts were investigated using in vitro study as *in silico* approach is reported to determine the nature of the plant phytochemicals interaction with the virulence proteins associated with MRSA in *Staphylococcus aureus.*

## 2 Materials and methods

### 2.1 Plant collection and extraction

*M. barbata* was collected from Dennieh (North Lebanon). Plant leaves and stems were dried and shredded via a laboratory shredder equipped with a 1 mm filter. Ground leaves and stems of were soaked in 70 % ethanol and left on a shaker at room temperature for 48 h. The filtrated crude extracts were collected and allowed to completely dry at room temperature. The stock solutions were prepared by dissolving the dried extracts in 10 % DMSO at 100 mg/ml concentration. The remain air-dried extract was kept in sterile bottles at room temperature. The sterility of the extract was tested before use by detection of any bacterial growth on Nutrient Agar after 24 h of incubation. The yield obtained from the stem extraction was 6.6 % compared to the leaves crude which was 5.6 %.

### 2.2 Extracts characterization

Fourier Transform Infrared Spectrometer (FTIR) and GC-MS analysis, were used to identify the chemical constituents of *M. barbata* extracts.

### 2.3 Fourier transform infrared spectrometer (FTIR)

The extracts were collected and dried prior to FT-IR measurement. The functional groups of the investigated extracts were detected via a Fourier Transform Infrared (FT-IR) spectrometer (Thermo Scientific Nicolet iS5 FT-IR). Spectra of the dried biomass of leaves and stem were recorded in the region of 400–4000 cm^−1^ with 32 scans.

### 2.4 Gas chromatography mass spectrometry analysis (GC-MS)

GC-MS Examination with gas chromatography-mass spectrometry (GC-MS), the components found in the ethanolic crude extracts of the stem and leaves were identified independently. SE and LE were each injected into a GC-MS connected to a mass spectrometer in a volume of 1 μl. After being kept at 80 C for 2 min, the temperature was raised to 250 C at a rate of 10 C per minute and maintained there for 5 min. The run took 30 min in all. Results from gas chromatography-mass spectrometry (GC-MS) were gathered using an Agilent 7890A device. Wiley NIST, a mass spectral reference library, was used to examine the peaks. Data were transmitted via the NIST library database as an additional tool to confirm the compound identity [6].

## 2.5 Molecular docking

### 2.5.1 Homology modelling

Both the RCSB PDB database (https://www.rcsb.org/) and the NCBI database (www.ncbi.nlm.nih.gov) were used to identify the key *S. aureus* proteins, which included CrtM (diapophytoene synthase), SarA (Accessory Regulator A), and the most visible proteins causing *S. aureus* and MRSA infections. The NCBI provided the amino acid sequence for every protein. Each protein's PDB codes in (Table 1) were taken out of the RCSB PDB database and downloaded. After downloading the 3D structure of each studied protein and modelling its primary structure, comparison between the obtained codes was performed to obtain the best PDB structure that matched the protein sequence with RMSD value equal 0 between reference and docked proteins.

**Table 1:** PDB codes and the important amino acids involved in the interaction of *S.aureus*.

Protein And PDB codes	Important Amino acids involved in the interaction	ΔG (clean geometry)	Crystal structure
SarA (2FNP)	GLN B 166, TYR B 162, LEU A 113, VAL A 116, PHE B 134, LEU B 160, ALA B 138, THR A 117, ASP A 120, ASN B 161, ILE A 106, LYS A 105, ILE A 103, THR A 104, MET A 115, LEU A 112, VAL A 116, LYS A 172, SER A 175, ASP A 181	2333.28 kcal/mol	
CrtM (2ZCO)	PHE A 22, TYR A 41, LEU A 164, VALA 137, LEU A 145, PHE A 26, LEU A 160, ILE A 241, GLY A 161, ALA A 134, LEU A 141, GLY A 138, ALA A 157, PHE A 267, LYS A 273, LYS A 20, LYS A 17, ARG A 265, SER A 19, HIS A 18, GLU A 264, VAL A 232, LYS A 231, LYS A 16, LYS A 13	1859.47 kcal/mol	

### 2.5.2 Selection and processing of the ligand structure

The major phytochemical compounds of *M. barbata* ethanolic crude extracts SE (Stem with ethanol) and LE (Leaves with ethanol), including palmitic, cyclohexane, pentanol, koumarone and safranal, were selected based on their available reported biological activity in the literature and the findings obtained from the GC– MS reports (Table 3, Table 4). The 3-D structure of the phytochemicals were retrieved from either Spider Chem or PubChem databases (Table 2). Geometric optimization was performed using Argus Lab 4.0.1 software. Quantum Mechanics (QM) method Austin model 1 (AM1) was used for refining the initial geometries using the “Clean Geometry” option in the ArgusLab.

**Table 2:** selected phytocompounds used in simulation process.

Phytochemical	Molecular Formula	IUPAC name	SMILES	3D structure
benzene	C_9_H_10_O	2-methoxyethenylbenzene	COC=CC1=CC=CC=C1	
Kumarone	C_11_H_14_O	7-methyl-4,4*a*,5,6-tetrahydro-3*H*-naphthalen-2-one	CC1=CC2=CC(=O)CCC2CC1	
Palmatic acid	C_16_H_32_O_2_	hexadecanoic acid	CCCCCCCCCCCCCCCC(=O)O	
Pentanol	C_9_H_16_O	5-(2-methylidenecyclopropyl)pentan-1-ol	C=C1CC1CCCCCO	
Safranal	C_10_H_14_O	2,6,6-trimethylcyclohexa-1,3-diene-1-carbaldehyde	CC1=C(C(CC=C1) (C)C)C=O	
Cyclohexane	C_10_H_14_O	2-methyl-6-propan-2-ylidenecyclohex-2-en-1-one	CC1=CCCC(=C(C)C)C1=O

**Table 3:** Identified phytochemical compounds in the crude extract of *M. barbata* stems using GC-MS.

Serial No.	CAS	Name of the Compound	Molecular Formula	Molecular weight g/mol	Retention Time	Peak Area (%)	Biological activity and REF
1	491-09-8	Piperitenone Cyclohexene (2-Cyclohexen-1-one, 3-methyl-6-(1-methylethylidene))	C_10_H_14_O	150.22	13.8	8.14	Antimicrobial activity [2]
2	57-10-3	Palmitic acid (hexadecanoic acid)	C_16_H_32_O_2_	256	22.4	22.9	Anti-inflammatory, antioxidant, anti-androgenic, hypocholesterolemic, hemolyticnematicide, pesticide, 5-α reductase inhibitor, potent mosquito larvicide, treat rheumatic symptom [11]
3	116-26-7	Safranal (2,6,6-trimethylcyclohexa-1,3-diene-1-carbaldehyde)	C_10_H_14_O	150.22	13.7	8.14	The therapeutic applications of saffron, which is linked to the presence of four major bioactive compounds: crocin, crocetin, picrocrocin, and safranal. Saffron has an antibacterial activity against a few clinical strains [33]
4	112-80-1	Oleic Acid	C_18_H_34_O_2_	282.255	22.4	22.9	Antifungal activity [1]

**Table 4:** Identified phytochemical compounds in the ethanol extract of *M. barbata* Leaves using GC-MS.

Serial No.	CAS	Name of the compound	Molecular Formula	Molecular weight (g/mol)	Retention Time	Peak Area (%)	Biological activity and REF
1	4747-15-3	Benzene, (2-methoxyethenyl)-	C_9_H_10_O	134.073	14.1	16.5	Not reported
2	–	1-Pentanol, 5-(methylenecyclopropyl)-	C_9_H_16_O	140.121	23.8	90.25	Not reported
3	34545-88-5	Kumarone 2(3H)-Naphthalenone, 4,4a,5,6-tetrahydro-7-methyl-	C_11_H_14_O	162.104	14.1	16.5	Not reported
4	–	Iso-menthone 2,4-Pentadien-1-ol, 3-pentyl-, (2Z)-	C_10_H_18_O	154.135	22.4	29	Not reported
5	112-80-1	Oleic Acid	C_18_H_34_O_2_	282.255	22.4	28.25	Antifungal activity [1]

### 2.5.3 Molecular simulation and docking studies

ArgusLab 4.0.1 was the software used for computational docking studies. Every crystal structure was ready for docking analysis by optimizing the H-bond and eliminating any water molecule to make easier the computations process and to clear the binding site from possible water molecules that can distort the search. Molecular Mechanics (MM) method was selected where the Universal Force Field (UFF) approaches was used. The grid dimensions of 2FNP protein with the selected SE and LE phytochemical compounds were 151 × 138 × 126 A^0^ and the grid dimensions of 2ZCO protein with the selected SE and LE phytochemical compounds were 151 × 145 × 151 A^0^ except for safranal compound was 139 × 136 × 149 A^0^. Using molecular dynamics modelling, interactions between the phytochemicals and *S. aureus* resistance proteins were investigated where the binding energy in Kcal/mol was computed to determine which of these compounds has the highest affinity for the targeted proteins. Furthermore, the docked complexes were visualized using BIOVIA Discovery Studio visualizer 2016 v16.1.0.15350 [28].

### 2.6 FTIR measurement of *S. aureus* treated with *M. barbata* ethanolic crudes

The interaction between *S. aureus* cells and ethanolic crude extracts was analyzed using Fourier Transform Infrared (FTIR) spectroscopy. Untreated bacterial cells in broth were used as a positive control and compared to *S. aureus* cells treated with (SE) and (LE) crudes at their respective (MIC) values. Two solutions were prepared for FTIR analysis: the first comprised untreated bacterial suspensions (10⁶ CFU/mL) in 5 mL of (MHB), while the second included bacterial suspensions (10⁶ CFU/mL) treated with SE and LE crudes at final concentrations of 12.5 mg/mL and 6.25 mg/mL, respectively. Both solutions were incubated at 37 °C for 24 h and subsequently centrifuged at 9,000 rpm for 20 min to isolate the bacterial pellets for FTIR analysis. FTIR measurements were performed on a Nicolet iS5 FTIR spectrometer equipped with a DTGS detector and KBr beam splitter, using transmission mode in the spectral range of 500–4000 cm⁻^1^. Spectra were recorded with a resolution of 4 cm⁻^1^ for bacterial biomass samples and collected over 64 scans against a ZnSe disc background. Each measurement was repeated three times for bacterial samples, ensuring reproducibility.

### 2.7 Microbial strains and growth conditions

*S. aureus* used in this study was obtained from an Islamic private hospital and maintained on Luria Broth (LB) medium at 37 ͦ C.

### 2.8 Antimicrobial activity

The antimicrobial activity of *M. barbata* crude extracts was determined by agar well diffusion assays. The bacterial suspension was set to 0.5 MacFarland (1.5 × 10^8^ CFU/mL) standard and then 100 μl were spread on Mueller-Hinton agar (MHA) plates. 20 μl of stem and leaves crude extracts suspended in 10 % Dimethyl sulfoxide (DMSO) were added aseptically to the wells with a final concentration of 100 mg/ml each. Plates were incubated at 37 °C for 24 and 48 h. DMSO was used as a negative control, imipenem (10 μg) was used as a positive control [12].

### 2.9 Determination of minimal inhibitory concentrations and minimum bactericidal concentration

Using the microtiter plate assay method, the minimum inhibitory concentration (MIC) of *M. barbara* extracts were determined. 100 μL of MHB was distributed in each well. The extracts compound was serially diluted 1:1 in 10 dilutions ranging from (100–0.195 mg/mL). *S. aureus* culture was added to each well to a final concentration of 1 × 10^6^ CFU/well. Medium alone were considered as the negative control, while medium inoculated with the tested strain was considered as the positive control. The MIC was recorded after 24 h of incubation at 37 °C and was defined as the lowest concentration that showed no growth [32]. Minimum bactericidal concentration (MBC) of SE and LE crude extract that did not exhibit any bacterial growth after inoculation of 10 μL of the mixture from wells that shows no turbidity on MHB, indicating 99.9 % killing of the original inoculum. The experiments were repeated in triplicate [23].

### 2.10 Biofilm inhibition assays

The effects of SE and LE crude extracts were investigated during biofilm development, a 10⁶ CFU/ml bacterial culture was added into each well of a 96-well culture plate with a flat bottom. After adding 150 μl crude extract ranging in concentration from 100 to 3.125 mg/ml, plates were aerobically incubated for 24 h at 37 °C. After incubation, the well contents were aspirated, washed three times with double-distilled water to remove planktonic cells, and the plates were dried in an inverted position. For the quantification of biofilm formation, each well was stained with 200 μL of 1 % crystal violet, incubated for 15 min at room temperature, rinsed twice with double-distilled water, and thoroughly dried. The remaining dye attached to the adherent cells was solubilized with 30 % (v/v) glacial acetic acid. After 30 min of incubation at room temperature, the total biofilm biomass was spectrophotometrically quantified at 490 nm [31]. The inhibition of biofilm formation was calculated by using the formula

$$\%\text{Biofilm}\;\text{inhibition}=\frac{{\text{OD}}_{\text{control}}‐\;{OD}_{\text{test}}}{{\text{OD}\;}_{\text{control}\;}}\times 100$$

where OD_control_ and OD_test_ is the absorbance without and with the addition of crude extracts, respectively.

### 2.11 Statistics

To ensure statistical confidence, each experiment was run three times. The experimental outcomes' data values were documented as the mean ± standard deviation. A one-way ANOVA was used to evaluate the significance, with a P-value ∗p < 0.05 were considered statistically significant. The statistical analyses were conducted using GraphPad Prism 10.1.0 (GraphPad Software Inc., San Diego, CA, USA).

## 4 Results and discussion

### 4.1 FTIR for leaves and stems extracts

**Fig. 1:**
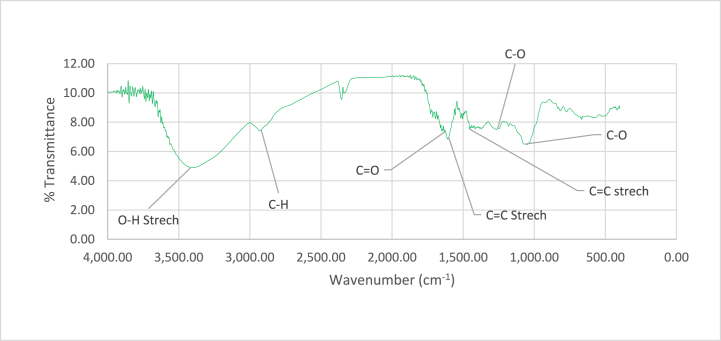
FTIR spectrum of leaves extract of *M. barbata*.

The LE crude material in FTIR spectra (Fig. 1) showed the bands that correspond to several functional groups. The strong broad peak value at 3625.16 cm^-^corresponds to the –OH stretching vibration observed in alcohol hydroxyl groups. A band of moderate strength at 2930 cm^−1^ is indicative of alkane group-specific C-H stretching vibration (alkanes). Furthermore, amide group-related C=O stretching vibrations are displayed by a separate peak at 1649 cm^−1^, while aromatic compound-specific C=C stretching vibrations are represented by bands at 1605.99 cm^−1^ and 1453.69 cm^−1^respectively. Vibrations identified in the fingerprint area (2000 cm^−1^to 500 cm^−1^), such as C=O amides, C=C stretching for aromatic compounds, C-O for carboxylic acids, and C-O amines determined the presence of these functional groups in the ethanolic extract of the plant leaf (Fig. 1).

**Fig. 2:**
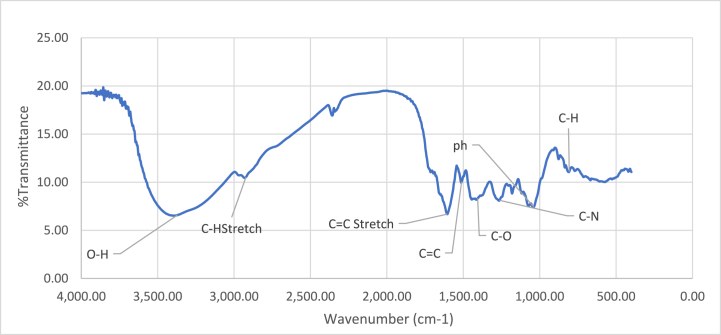
FTIR spectrum of stems extract of *M. barbata*.

Likewise, the SE crude's FTIR spectrum (Fig. 2) shows its characteristic peaks with different vibrational modes. Strong broad band at 3393.96 cm^−1^ correspond to O-H group indicate the presence of organic compounds in the extract. The peak at 2930.8 cm^−1^ suggests C-H stretching vibrations, and indication of interacting alkane group. Specifically, a peak at 1605.05 cm^−1^ indicated the existence of an aromatic molecule containing C=C bond. Several characteristic peaks are seen in the fingerprint area (1513.20 cm^−1^ to 500 cm^−1^), including -CH_3_ bending, C-O, C-N amines and C-H bending, vibrations. These apparent differences and similarities in the FTIR spectra imply that although the two foundational components share comparable chemical characteristics, such as aromatic and alkane molecules, they have different compositions with various functional groups. That particular chemical profiles may imply varied applications or antimicrobial potentials against *S. aureus*.

### 4.2 GC-MS analysis

The GC-MS data provides valuable insights into the phytochemical composition of *M. barbata* stems and leaves, allowing for the identification and comparison of various compounds based on their retention times, peak areas, and mass spectral fragmentation patterns**.** The ethanol extracts of *M. barbata* leaves and stem were characterized using the GC-MS equipment. The results of the *Micromeria* extracts/parts chromatograms are displayed in Table 3, Table 4

Nine distinct compounds were identified using GC-MS analysis of ethanol extracts from the stems and leaves of *M. barbata*. Four compounds with peak areas ranging from 8.14 % to 22.93 % were detected in the stem extract: piperitenone cyclohexene, palmitic acid, safranal, and oleic acid. Notably, the retention time of the identified palmitic saturated fatty acid was 22.41 which was previously reported in family Lamiaceae [24], but the retention time of piperitenone cyclohexene was 13.827. Oleic acid was identified as unsaturated fatty acid showed a retention time of 22.38 and safranal exhibited a retention time of 13.79. On the other hand, the leaf extract included five different chemicals that were detected, including iso-menthone, oleic acid, 1-pentanol, kumarone and benzene. Among these, iso-menthone and oleic acid were also present with same retention times of 22.41, some studies reported similar findings the most abundant compounds in all samples and extracts of *Micromeria fruticosa* were menthone, menthol, oleamide, and pulegone [9]. The most remarkable compound was 1-pentanol, 5-(methylenecyclopropyl), which had a notable peak area of 90.25 % and a molecular weight of 140.121 g/mol with a retention time of 23.86 which correlates with the presence of alcohol hydroxyl groups presented by O-H functional groups in FTIR findings. Kumarone and benzene were detected also at the same retention time with similar abundancy 16.5 % in the LE extract.

These results identify the unique phytochemical profiles of the stems and leaves of *M. barbata*, offering important new information on their possible biological activities and medicinal properties. Furthermore, most of the previous research on *M*. *barbata* focused primarily on its essential oil [3]; no studies had ever been conducted on the GC-MS analysis of its crude extract. The identification of compounds such as piperitenone, cyclohexene, and safranal in the GC-MS analysis correlates with the presence of functional groups related to aromatic compounds shown in the FTIR spectra, indicating the existence of aromatic molecules in both extracts. Overall, the differences in the identified compounds, their respective retention times, and peak areas highlight the distinctive phytochemical profiles of the stem and leaf extracts from *M. barbata*.

### 4.3 Molecular docking simulation of *S.aureus* virulence proteins

The findings of the molecular docking analysis, as depicted in Table 5, Table 6, illustrate the minimized energy values (in kcal/mol) of *M. barbata* phytochemical compounds interacting with SarA and CrtM proteins of MRSA. The docking interactions were specifically targeted at 2FNP and the CrTM active sites. Through this analysis, distinct binding affinities were revealed between the two proteins, 2ZCO (CrTM) and 2FNP (SarA), towards the various ligands.

**Table 5:** Molecular docking analysis reveals stem ethanolic bioactive compounds’ binding energy with SarA and CrtM of MRSA.

SE compound	IUPAC name	2ZCO (CrTM)		2FNP (Sar A)	
		Binding energy (Kcal/mol)	Ki Inhibition constant (μM)	Binding energy (Kcal/mol)	Ki Inhibition constant
Safranal	2,6,6-trimethylcyclohexa-1,3-diene-1-carbaldehyde	−7.7	22.64 μM	−10.5	20.15 nM
Cyclohexene	2-methyl-6-propan-2-ylidenecyclohex-2-en-1-one	−8.37	73.12 μM	−10.41	23.4Nm
Palmitic acid	hexadecanoic acid	−7.1	68.5 μM	−11.1	73.3 nM

**Table 6:** Molecular docking analysis reveals leaves ethanolic bioactive compounds’ binding energy with SarA and CrtM of MRSA.

LE compound	UPAC name	2ZCO(CrTM)		2FNP(SarA)	
		Binding energy (Kcal/mol)	Ki Inhibition constant	Binding energy (Kcal/mol)	Ki Inhibition constant
Kumarone	7-methyl-4,4*a*,5,6-tetrahydro-3*H*-naphthalen-2-one	−7.9	16.25 μM	−11.3	5.22 nM
Benzene	2-methoxyethenylbenzene	−6.5	17.2 μM	−10.9	10.31 nM
Pentanol	5-(2-methylidenecyclopropyl)pentan-1-ol	−7.8	19.14 μM	−11.6	3.13 nM

Among the SE tested compounds, palmitic acid, cyclohexane, and safranal exhibited stronger interactions with 2FNP (SarA), with binding energy values of −11.1, −10.41, and −10.5 kcal/mol, respectively. In contrast, interactions with 2ZCO (CrTM) showed slightly lower binding energies, with values of −8.37, −7.7, and −7.1 kcal/mol for cyclohexane, safranal, and palmitic acid, respectively. This consistent pattern of stronger binding for 2FNP (SarA) suggests potential differences in the binding pocket forms, amino acid residues, or general protein-ligand interactions between the two proteins. Binding affinity values, generated through molecular docking, signify the strength of association between two molecules and reflect the spontaneous reaction occurring at the targeted protein's active site. This reaction is closely correlated with the minimized energy resulting from the disruption of total intermolecular and torsional energies [28].

Moreover, when considering LE crude molecules such as kumarone, benzene, and pentanol, 2FNP (SarA) consistently displayed higher binding energies compared to 2ZCO (CrTM). Specifically, interactions with kumarone (−11.3 kcal/mol vs. −7.9 kcal/mol), benzene (−10.9 kcal/mol vs. −6.5 kcal/mol), and pentanol (−11.6 kcal/mol vs. −7.8 kcal/mol) were notably stronger with 2FNP (SarA) which correlate with the results of the ki values presented in Table 4, Table 5 showing that M. barbata crudes were exclusively more potent on SarA virulence proteins of the *S. aureus* than the CrTM virulence proteins and mainly LE ethanolic bioactive compounds were more potent inhibitors of the targeted virulence proteins showing the lowest ki as the lowest concentration of the compound needed to inhibit the target proteins.

**Fig. 3:**
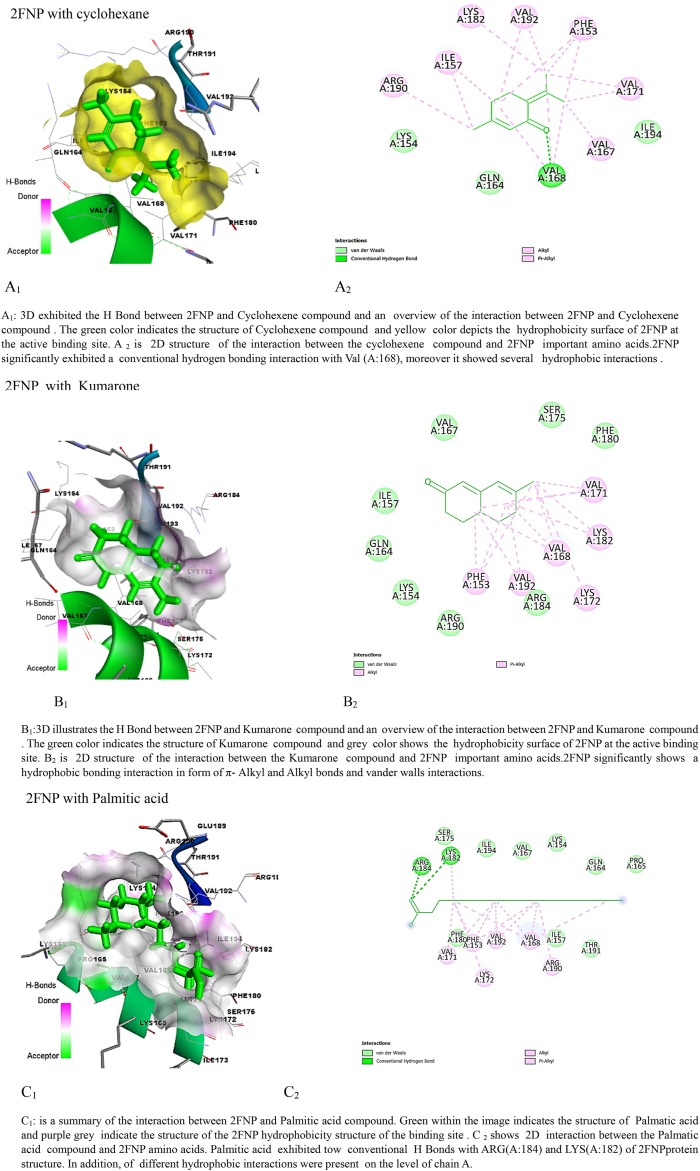

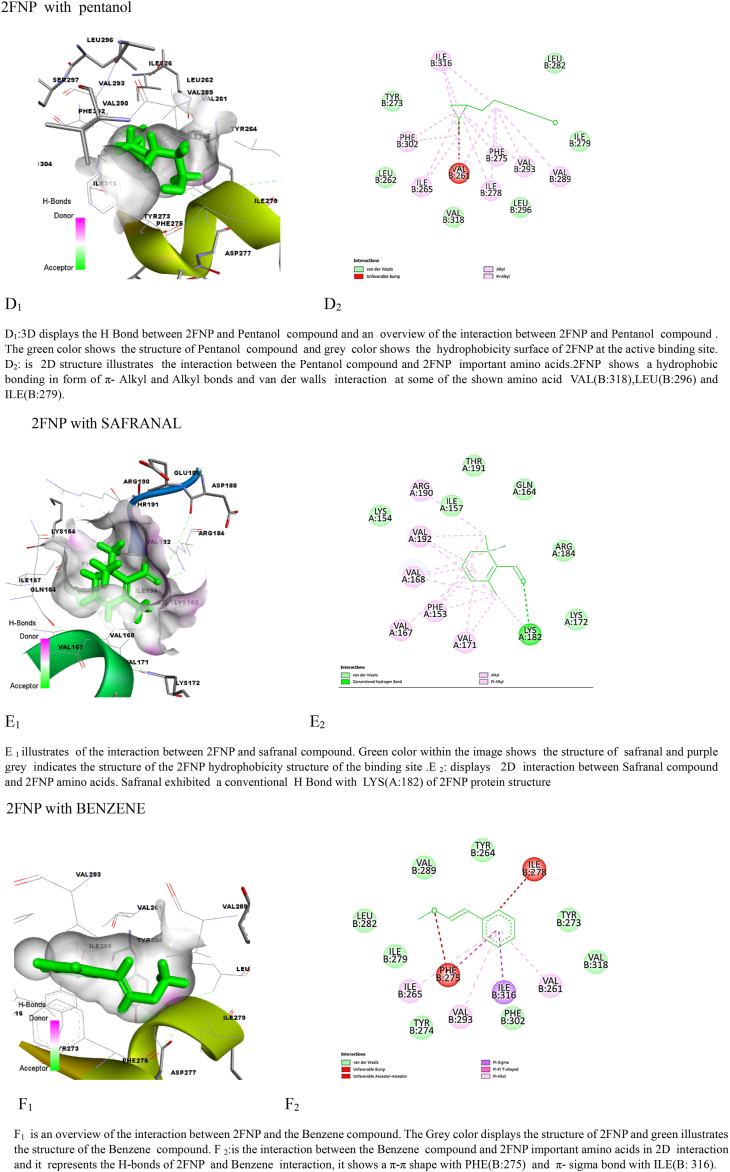

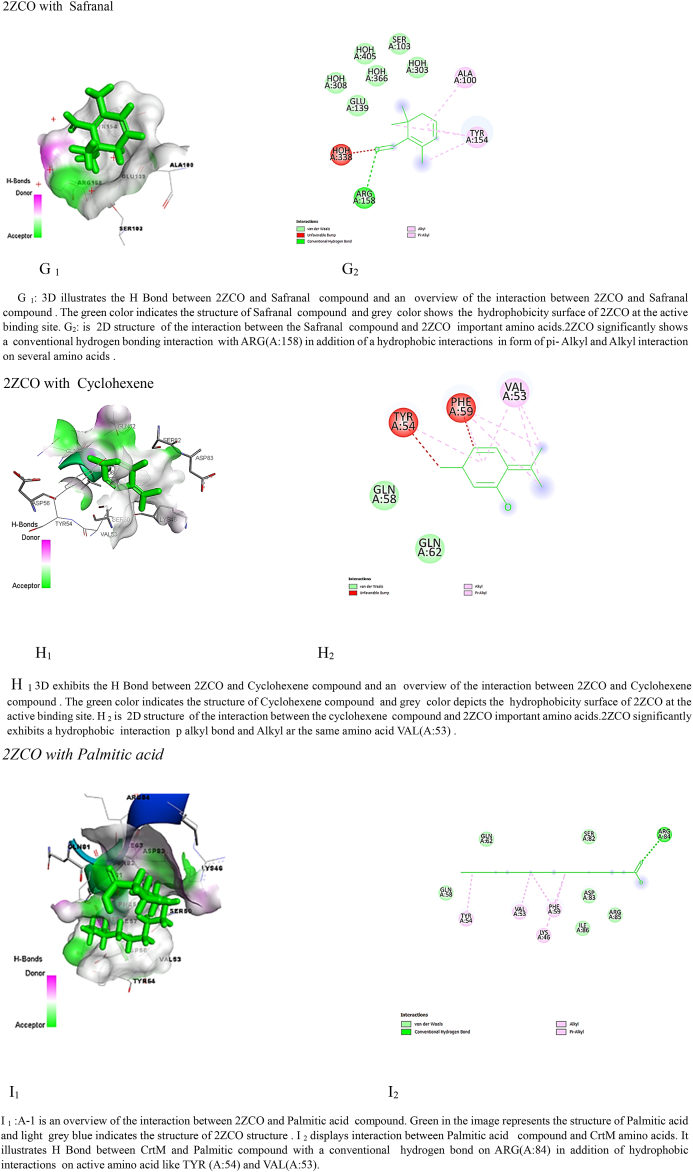

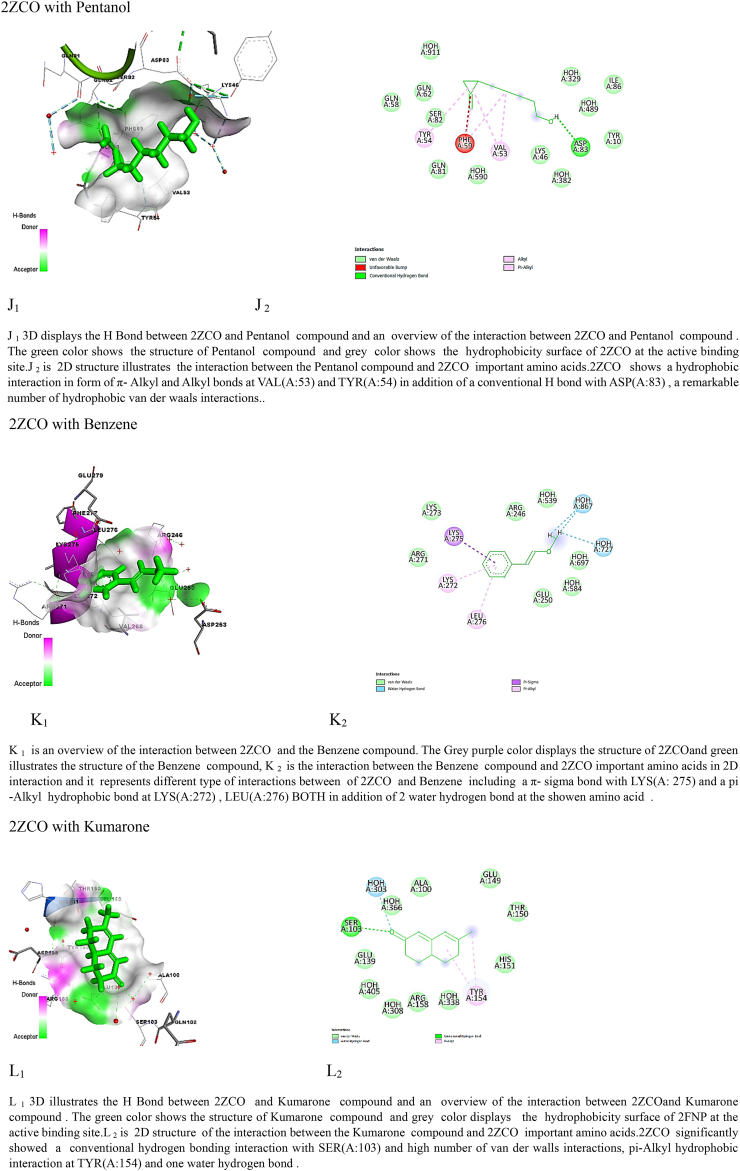
Molecular docking analysis: two-dimensional (2D) and three-dimensional (3D) representation of interaction patterns of the selected com pounds and SarA and CrtM proteins. Left panel: 3D interaction among compounds with SarA of MRSA. Right panel: 2D diagram interaction between active sites of (SarA and 2ZCO) and the identified compounds.

Hydrogen bonding, a key feature observed in multiple interactions in Fig. 3 (A1, B1, C1, D1, E1, G1, H1, L1), plays a crucial role in stabilizing ligand-protein complexes by forming directional bonds between specific amino acids of the proteins and the compounds. Additionally, hydrophobic interactions, depicted by π-alkyl and alkyl bonds as well as van der Waals interactions, contribute significantly to complex stability, as evident in interactions with 2FNP Fig. 3 (A1, B1, C1, D1, E1) and 2ZCO Fig. 3 (H1, J1, I1, K1, L1), these interactions contribute to the stability of the ligand-protein complex at the active site of CrtM.

The specific amino acid interactions vary across different compounds, with some amino acids interacting more prominently with certain ligands. Supramolecular interactions, such as π-π interactions with PHE(B:275) and a π-sigma with ILE (B: 316), further enhance binding affinity and are crucial in drug design and molecular recognition processes [28].

While 2FNP and 2ZCO both show identical types of interactions with the molecules, there may be differences in the particular amino acids that are involved and the type of interactions. As a case study, 2ZCO primarily interacts with amino acids ARG, ASP, and SER, whereas 2FNP primarily interacts with Val, ARG, and LYS. These findings provide significant information on the ligand-binding preferences of 2FNP (SarA) compared to 2ZCO (CrTM) and suggest possible questions regarding the molecular mechanisms behind these interactions.

### 4.4 FTIR measurement of *S. aureus* treated with *M. barbata* ethanolic crudes

**Fig. 4:**
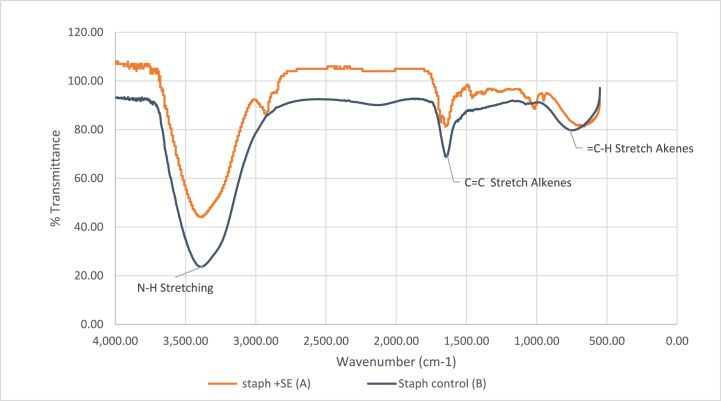
(**A**) FTIR spectrum from *S. aureus* treated by ethanolic Stem extract of *M. barbata* (**B**) FTIR spectrum from *S. aureus* control.

**Fig. 5:**
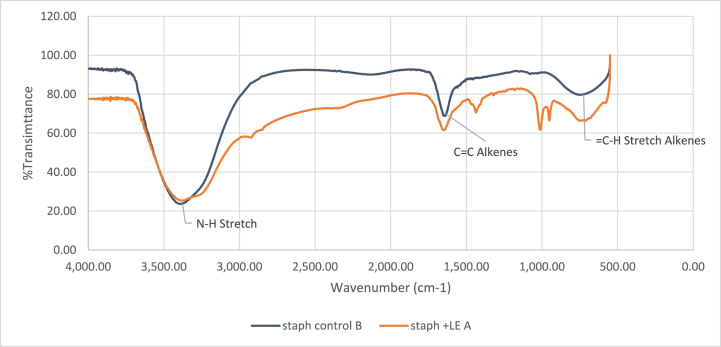
(**A**) FTIR spectrum from *S. aureus* treated by ethanolic Leaves extract of *M. barbata* (**B**) FTIR spectrum from *S. aureus* control.

FTIR spectroscopic analysis revealed significant alterations in the spectral profiles of both stem and leaf ethanolic crude-treated *S. aureus* cells compared to untreated cells. Fig. 4, Fig. 5 A,B illustrate these changes. The spectra displayed variations in bands ranged between 4000 and 3000 cm^−1^, indicative of modifications in membrane phospholipids and polysaccharides, highlighting the impact of *M. barbata* ethanolic crude on *S. aureus* membrane integrity. In both ethanolic crude samples, the band associated with phospholipid components of *S. aureus* cell membrane at 3440.2 cm^−1^ exhibited reduced intensity compared to untreated cells [8]. Furthermore, differences were observed in the 3000–2000 cm^−1^ region between the two spectra of SE and LE crudes. Specifically, SE-treated *S. aureus* cells displayed a blue shift in the fatty acid peak with an O-H shoulder at 3330 cm^−1^, while LE-treated cells exhibited a blue shift at 287.3 cm^−1^ in the same region, indicating alterations in the biological material's characteristics. The spectral regions between 500 and 3500 cm^−1^, corresponding to proteins, lipids, and nucleic acid backbone signals, demonstrated notable changes. Additionally, a blue shift was noted in the protein peaks within the 2000-1500 cm^−1^ range, suggesting modifications in protein structure. Furthermore, changes in the peak attributed to nucleic acids at 1000 cm^−1^ were observed in both spectra following incubation of *S. aureus* with *M. barbata* ethanolic crudes.

### 4.5 Antimicrobial activity of extracts

**Fig. 6:**
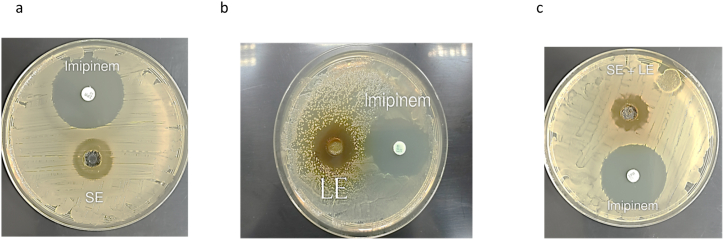
A SE:Stem + Ethanol (IZ = 15 mm); Fig. 6-b LE: Leaves + Ethanol (IZ = 20 mm) with 100 mg/ml for each crude; Fig. 6-c: SE + LE:Leaves + Stem (IZ = 22 mm), Imipenem (IZ = 25 mm).

In the current investigation, the antimicrobial properties of the crude extracts from the stems and leaves of *M. barbata* were evaluated. Utilizing a concentration of 100 mg/ml, the extracts’ efficacy was tested against *S. aureus* via the agar well diffusion method. The results, depicted in Fig. 6, indicate that the stem extract exerted a modest inhibitory effect on *S. aureus*, producing an inhibition zone measuring 15 mm (Fig. 6a). In contrast, a synergistic blend of stem and leaf extracts manifested moderate antibacterial activity (Fig. 6c). Notably, the leaf extract alone was found to be the most potent, inhibiting bacterial growth with an inhibition zone of 20 mm (Fig. 6b). When benchmarked against the standard antibiotic Imipenem (10 μg), which yielded an inhibition zone (IZ) of 25 mm, the activities of the tested crude extracts were deemed moderate. This aligns with the findings of Al-Hamwi et al. (2015) [15], which highlighted the significant antibacterial activity of the phenolic constituents in *M. fruticosa* extract, evidenced by a 30 mm inhibitory zone against *S. aureus*. The burgeoning interest in the application of herbal antibiotics to combat *S. aureus* infections has been underscored by recent studies [23]. A plethora of research has affirmed the antibacterial virtues of the Micromeria genus. Among the species studied, *M. fruticosa* has been the subject of extensive pharmacological scrutiny [7], with other notable species including *M. cilica*, *M. barbata*, *M. Juliana*, *M. thymifolia*, *M. cristata*, *M. croatica*, *M. myrtifolia*, *M. nervosa*, *M. albanica*, and *M. persica* demonstrating significant pharmacological activities.

### 4.6 Minimum inhibitory concentration (MIC) and minimum bactericidal concentration (MBC)

There are no generally accepted standard MIC end-points for in vitro testing of plant extracts. However, Kuete in 2010 proposed that the antibacterial activity of a plant extract is considered significant when MIC values are below 0.1 mg/ml, moderate when 0.1 ≤ MIC≤ 0.625 mg/ml and weak when MIC >0.625 mg/ml [16]. According to Kuete's proposed classification, the study categorizes the ethanol extract of *M. barbata* leaves as moderately potent (MIC = 6.25 mg/ml) against *S. aureus*, while the stem ethanol extract demonstrates weaker activity (MIC = 12.5 mg/ml) results are shown in Table 7. These results align with the agar well diffusion test, providing consistency in the assessment of antimicrobial properties. The MBC/MIC ratio shown in Table 7, employed to discern between bacteriostatic and bactericidal effects, indicates that both ethanol extracts exhibit bactericidal activity against *S. aureus*, as the MBC values are found to be lower than the corresponding MIC values. An MBC/MIC ratio less than 4 is generally interpreted as bactericidal, emphasizing the potential of Micromeria extracts to kill rather than inhibiting bacterial growth [13,20]. Comparisons with previous studies on the Micromeria genus, particularly *M. fructicosa*, reinforce the current findings. The documented antibacterial action of the phenolic extract of Micromeria, with a MIC value of 0.375 g/ml, and notable antifungal activity against Candida albicans provide additional context to the potential therapeutic applications of these plant extracts [15]. The observed variations in bacterial susceptibility among studies may be attributed to inherent differences in microbial tolerance or the diverse physico-chemical properties of phytochemicals present in the crude extracts [18].

**Table 7:** MIC and MBC of crude extracts on *S. aureus*.

*Micromeria* ethanolic crude	MIC (mg/ml)	MBC (mg/ml)	MBC/MIC ratio
LE crude	6.25 ± 1.5	12.5 ± 1.5	2 bactericidal
SE crude	12.5 ± 3.5	25 ± 3.5	2 bactericidal

These collective findings contribute to the growing body of knowledge regarding the biological activities of Micromeria species [7]. While *M. fructicosa* has been extensively studied, this investigation sheds light on the antimicrobial potential of *M. barbata*, particularly its leaves and stems. The moderate to weak antibacterial activities against *S. aureus*, along with bactericidal effects, underscore the promising nature of Micromeria extracts in combating bacterial infections. Further research could delve into the specific phytochemical composition responsible for these activities and explore potential applications in the development of novel antimicrobial agent.

### 4.7 Biofilm formation assays

The ability of *M. barbata* extracts to inhibit biofilm formation of MRSA was evaluated by crystal violet staining biofilm quantification assay in 96 well polystyrene plates. The result showed a concentration -dependent antibiofilm potential of crudes against *S. aureus.*

**Fig. 7:**
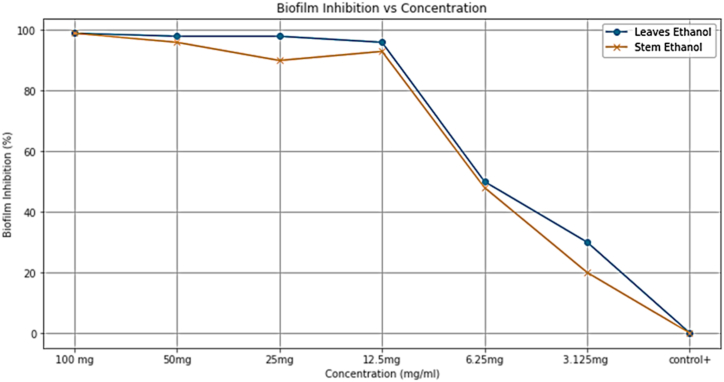
Antibiofilm activity of crude extract against *S. aureus*.

LE and SE crudes at 100 mg/ml concentration showed a maximum inhibition of 99 %, compared to SE with % of biofilm inhibition in *S. aureus* at the same concentration value. At 50 mg/ml concentration LE crude exhibited 98 % which was higher value than SE crude with 95 % as antibiofilm activity. Both crude showed % of biofilm inhibition <50 % for 6.25 mg/ml,3.125 mg/ml concentrations. Thus the present study revealed the increase in biofilm inhibition with an increase in Ethanolic crude concentration. The results of anti-biofilm (ABF) potential of the plant extracts against *S. aureus* are presented in Fig. 7.

These results supports the idea that anti biofilm activity's dependent on concentration and highlights an advantage for using LE crude at this concentration. Extracts resulting in inhibition above 50 % were considered to have good antibiofilm activity while those with inhibition between 0 and 50 % indicated poor ABF activity, and the values < 0 were regarded as no inhibition, or enhancement of biofilm development and growth [25].

## 5 Conclusions

Constituents of LE and SE ethanolic extract of *M. barbata* obtained from FTIR & GC-MS showed an antimicrobial activity against MRSA. Molecular docking simulations suggested potential bioactivity of the identified phytochemical compounds by GC-MS against *S. aureus* virulence proteins, supported by antimicrobial assays where leaf extract (LE) showed higher antimicrobial activity with (MIC: 6.25 mg/ml) compared to stem extract (SE) with MIC of 12.5 mg/ml. From the two extracts, both displayed good antibiofilm activity against *S. aureus*, which has demonstrated its ability to inhibit biofilm formation up to a reduction level of 90 %.This study indicates a comprehensive understanding of the phytochemical composition and biological activities of the *M. barbata* extracts, offering valuable insights into their potential applications as antimicrobial and antibiofilm agents**.**

## Data availability

All data generated or analyzed during this study are included in this published article and the attached supplementary file.

## CRediT authorship contribution statement

**Shiraz Rawas:** Visualization, Software, Resources, Project administration, Methodology, Investigation, Formal analysis, Data curation. **Nawal Al Hakawati:** Writing – review & editing, Supervision, Data curation. **Amale Mcheik:** Data curation. **Dalia El S El Badan:** Supervision, Methodology, Formal analysis.

## Declaration of competing interest

The authors declare that they have no known competing financial interests or personal relationships that could have appeared to influence the work reported in this paper.
