# Supplementary figures and images for: Micromeria barbata for targeting MRSA virulence: In silico and in vitro studies
Source: Heliyon. 2024 Dec 29;11(1):e41536. doi: 10.1016/j.heliyon.2024.e41536 (PMC11754165; doi:10.1016/j.heliyon.2024.e41536)

Supplementary Figures


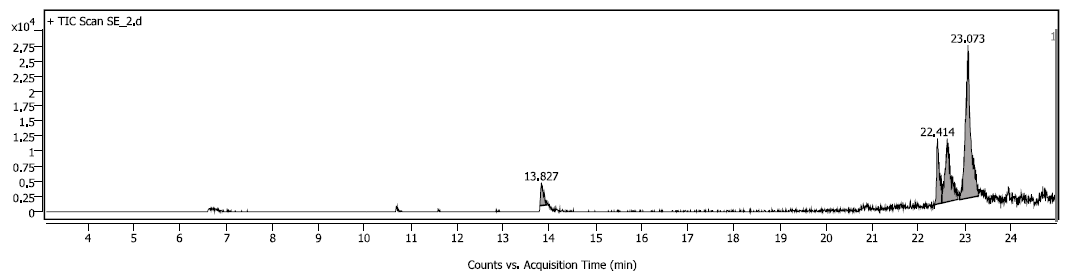


Figure A: GC/MS SE Crude


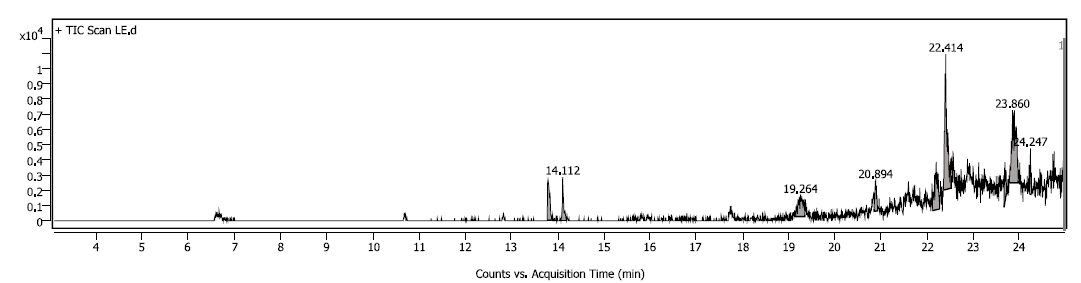


Figure B: GC/MS LE Crude

Supplement: Multimedia component 1 [file mmc1.docx]
